# Bushen Yijing Decoction (BSYJ) exerts an anti-systemic sclerosis effect via regulating MicroRNA-26a /FLI1 axis

**DOI:** 10.1080/21655979.2021.1907128

**Published:** 2021-04-12

**Authors:** Zixuan Cheng, Jialin Zhang, Wanying Deng, Shaojian Lin, Donghai Li, Ke Zhu, Qing Qi

**Affiliations:** a Department of Dermatology, The First Affiliated Hospital, Guangzhou University of Chinese Medicine, Guangzhou, China; b Department of Dermatology, The First Affiliated Hospital, School of Clinical Medicine of Guangdong, Pharmaceutical University, Guangzhou, China; c Department of Dermatology, Sun Yat-Sen Memorial Hospital, Sun Yat-Sen University, Guangzhou, China; d Department of Dermatology, The Second Affiliated Hospital, Guangzhou Medical University, Guangzhou, China

**Keywords:** miRNA-26a, dermal fibrosis, FLI1, systemic sclerosis skin fibroblast, Bushen Yijing decoction

## Abstract

Systemic sclerosis (SSc) refers to a group of autoimmune rheumatic diseases. Bushen Yijing decoction (BSYJ) is used for treating SSc. However, its underlying mechanism remains unknown. The present study aims to investigate potential roles of Friend leukemia integration factor 1 (FLI1) and microRNA in the beneficial effects of BSYJ on SSc. Primary skin fibroblasts were isolated from healthy individuals and SSc patients through tissue-explant technique and validated by immunocytochemistry. mRNA and microRNA levels were determined by quantitative RT-PCR. Protein expression was measured by western blotting. MiR-26a mimics or inhibitor were transfected to induce miR-26a overexpression or knockdown in vitro and in vivo, respectively. Histological changes of skin tissues from SSc mouse were evaluated by H&E and Masson trichrome staining. Results showed that FLI1 expression significantly decreased in primary skin fibroblasts of SSc patients. MiR-26a was predicted to target FLI1 untranslated region. Transfection of miR-26 mimics in SSc skin fibroblasts (SFB) leads to decrease in FLI1 expression and increase in collagen I gene expression and fibronectin accumulation. On the other hand, miR-26a knockdown increased FLI1 expression and decreased collagen I and fibronectin expression in SFB. In addition, BSYJ-containing rat serum suppressed miR-26a expression, while it elevated FLI1 expression and inhibited fibronectin and collagen I accumulation in SFB. In the mouse SSc model, BSYJ-containing serum inhibited dermal fibrosis by suppressing miR-26a expression and restoring FLI1 protein levels. Overall, our study demonstrates that BSYJ decoction exerts anti-dermal fibrosis in SSc patients via suppressing miR-26a level and thus to increase FLI1 expression in fibroblasts.

## Graphical abstract

**Figure:**
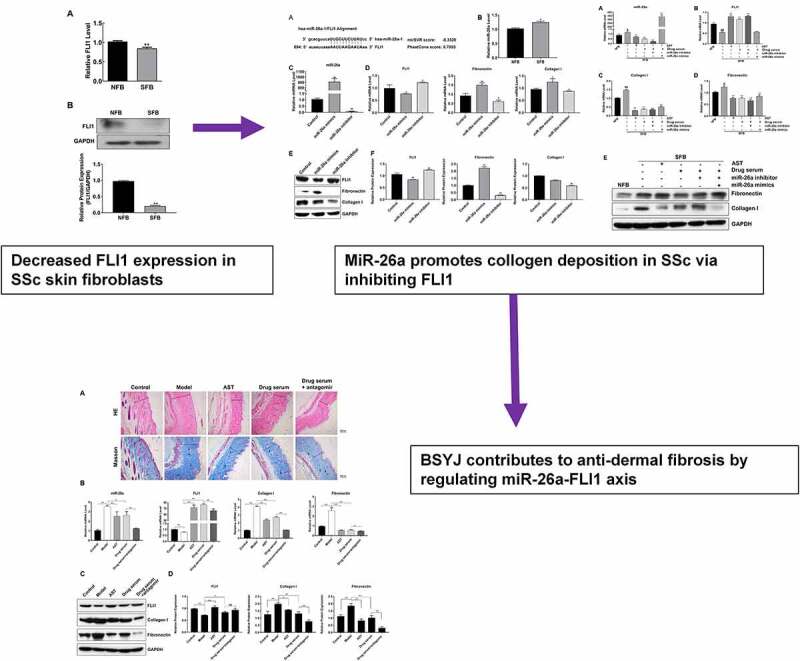

## 1. Introduction

Systemic sclerosis (SSc) is autoimmune rheumatic disease characterized by vascular injury and inflammation, autoantibody production, fibrosis, and pathological remodeling of connective tissues (usually in the skin, muscles, and other visceral organs) [1,2]. The pathophysiology of SSc features the architectural disruption of tissues due to uncontrolled myofibroblasts and excessive deposition of extracellular matrix (ECM) proteins, such as collagen type I [2,3]. The pathogenesis of SSc involves the persistent activation and proliferation of resident fibroblasts, which are not cleared as would occur during wound healing processes. This produces a microenvironment which is rich in ECM proteins and growth factors such as fibroblast growth factor (FGF) and connective tissue growth factor (CTGF) [2]. Friend leukemia integration factor 1 (FLI1) is a regulator of collagen fibrillogenesis and vascular homeostasis. Downregulation or deficiency of FLI1 is associated with collagen expression and SSc vasculopathy [4,5]. However, the molecular mechanisms responsible for FLI1 insufficient expression in SSc pathogenesis remain unclear.

Methotrexate, mycophenolate mofetil and cyclophosphamide are currently used to improve SSc patients’ symptoms, but the therapeutic option for this disease is limited. However, increasing evidence showed that traditional Chinese medicine (TCM) provides beneficial effects on SSc. For instance, Bushen Yijing formula could relieve dermal fibrosis in the mouse model of SSc [6]. The effects of Bushen Yijing on SSc fibroblast phenotype are potentially related to the SMAD3/FLI1 axis in the TGF-β pathway [7]. Moreover, the protective effects of Bushen Yijing on SSc may be through inhibiting occlusive vascular injury with fiber hyperplasia and regulating Snail-mediated EndMT [8]. TCM formula of Bushen Yijing therapy interacted Fli1’s expression to inhibit EndMT [9]. Overall, the above studies demonstrate Bushen Yijing therapy protects against SSc. Bushen Yijing therapy(BSYJ) based on symptom differentiation is widely used for osteoporosis systemic treatment, senility regulation, and SSc [10–12]. However, its underlying molecular mechanism remains unclear.

MicroRNA (miRNA) is a large group of small non-coding RNA molecules consisting of 22 nucleotides, which are widely expressed in various species and post-transcriptionally suppressed, and they regulate gene expression by directly targeting at 3'-untranslated region (UTR) [13,14]. Recent investigation has revealed that miRNA expression is closely associated with the pathogenesis of fibrosis [15]. In addition, epigenetics also plays important roles in SSc [16,17,18]. A large number of miRNAs, such as miR-146, miR-503, and miR-145, are differentially expressed in limited cutaneous SSc and diffuse cutaneous SSc tissues [19]. Specifically, miR-21 was identified as a key regulator in the TGF-β signaling events during fibrosis in SSc, and downregulation of miR-7 was responsible for excessive collagen deposition in localized SSc [20,21]. Moreover, miR-196a expression was significantly lower in SSc tissues, leading to increased type I collagen protein expression during SSc pathogenesis [22]. The above studies elucidate the significant roles of miRNAs in the development of SSc.

MiR-26a was reported to be involved in various fibrosis processes, including cardiac fibrosis and idiopathic pulmonary fibrosis [23,24]. However, it is largely unknown about its expression and functions in the pathogenic processes of SSc. In the present study, we investigated the effects of miR-26a and its predicted target gene, FLI1, on dermal fibrosis in SSc patients, and their roles in the protective effects of BSYJ on dermal fibrosis using SFBs and a mouse model of SSc. The present study provided novel insights into the pharmaceutical roles of BSYJ on SSc.

## 2. Materials and methods

### 2.1 Fibroblast primary culture

Primary skin fibroblasts were isolated from the skin tissues of SSc patients and healthy volunteers in the First Affiliated Hospital of Guangzhou University of Chinese Medicine using a tissue-explant technique. The tissue collection and following operations were approved by the Ethics Committee of Guangzhou University of Chinese Medicine, and informed consent was provided by each patient and volunteer. Briefly, four systemic sclerosis (SSc) patients and four healthy individuals were recruited. As shown in Table 1, all SSc patients were female, and one out of four patients were diagnosed with diffused subtype of SSc. The rest of patients were diagnosed with limited subtype of SSc. The normal control cohorts were male. Serum antinuclear antibody (ANA) of all the patients was positive. Among patients, one patient was positive in serum autoantibody (SCL-70) test, one patient was anti-centromere antibody (ACA) positive, and one patient was anti-SSA, anti-Sm, anti-U1-rRNP and dsDNA positive (Table 1). The skin tissues collected during surgical operations were repeatedly washed with phosphate-buffered saline (PBS) solution containing 100 U/mL penicillin and 100 U/mL streptomycin in petri dishes until they appeared pale and mildly swollen. The skin tissues were then cut into 1 × 1 cm pieces, which were then transferred into 25 cm^2^ cell culture flasks using sterile forceps. After incubation at 37°C for 2 h under 5% CO_2_, the skin tissue biopsies in the cell culture flasks were immersed in high-glucose DMEM culture medium (Thermo Fisher Scientific) containing 10% fetal bovine serum (FBS), 100 U/mL penicillin and 100 U/mL streptomycin. The volume of cell culture medium was strictly controlled to prevent the skin tissue from floating away from the bottom of the cell culture flask. After 24 h culture, the tissue biopsies were further incubated with an equal volume of complete cell culture medium. The cell growing status was then observed every 2 days. Primary human skin fibroblasts were identified by immunocytochemistry.

**Table 1.** The characteristic of SSc patients and control cohorts

	Age	Sex	Group	Subtype	Site of specimen	Course (y)	Therapy*	Antibody	ILD*
1	42	Female	SSc	Limited cutaneous type	Right forearm	1	Glucocorticoid	ANA	0
2	49	Female	SSc	Limited cutaneous type	Left forearm	1	Glucocorticoid; cycloposphamide	ANA; Anti-Centromere Antibody	0
3	23	Female	SSc	Limited cutaneous type	Right forearm	2	None	ANA; anti-SSA; anti-Sm; anti-U1-rRNP; dsDNA	1
4	47	Female	SSc	Diffuse cutaneous type	Right forearm	1	None	ANA; Scl-70	1
5	19	Male	Normal		Foreskin				
6	26	Male	Normal		Foreskin				
7	27	Male	Normal		Foreskin				
8	20	Male	Normal		Foreskin				

Therapy*: Immunosuppressive therapy or glucocorticoid therapy; ILD*, interstitial lung disease: 1: Yes; 0: No.

### 2.2 Immunocytochemistry (ICC) assay

Coverslips were coated with polyethyleneimine for 1 h at room temperature and rinsed three times with ddH_2_O for 1 h. The coverslips were used for cell growth after being completely dried and sterilized under UV light for 3 h. For ICC, the coverslips were briefly washed with PBS solution, fixed with 4% paraformaldehyde, washed three times with ice-cold PBS, permeabilized with PBS solution containing 0.2% Triton X-100 for 10 min, and washed three times with PBS solution for 5 min. Subsequently, cells in coverslips were blocked followed by incubation with anti-cytokeratin (CK) primary antibody (#SC374386; Santa Cruz, 1:100) and anti-vimentin primary antibody (#PB9359; Boster, 1:200) overnight at 4°C. Afterward, the samples were washed with PBS solution and then incubated with secondary antibodies (Boster, 1:500) diluted in 1% BSA for 1 h at room temperature. After counterstaining with DAPI (DNA stain) for 1 min, the coverslips were finally rinsed with PBS, mounted with one drop of mounting medium, and observed under microscopy.

### 2.3 Quantitative RT-PCR

Relative levels of mRNAs and miRNAs in cultured cells and mouse tissues were analyzed by a quantitative real-time (RT)-PCR. Briefly, RNA samples were extracted from cultured cells and mouse tissues using the TRIzol reagent (#15596026; Thermo Fisher Scientific) in accordance with the manufacturer’s instructions. The miScript II RT Kit (#218160; Qiagen) was used for cDNA synthesis by reverse transcription of total RNA containing miRNA. The relative expression levels were quantitatively assessed by RT-PCR using the RT2 SYBR® Green qPCR Mastermixes (#330500; Thermo Fisher Scientific) following the manufacturer’s instructions. The PCR reaction parameters were as follows: pre-denaturation for 2 min at 95°C, followed by 40 cycles of denaturation for 20 s at 94°C, annealing for 20 s at 58°C, and extension for 20 s at 72°C. Expression levels were finally quantified through the 2^−ΔΔCt^ method. Glyceraldehyde-3-phosphate dehydrogenase (GAPDH) and U6 were applied as the internal standards. At least three biological repeats were performed for statistical analysis to determine significant differences. The sequences of the primers used in quantitative RT-PCR in this study are listed in Table 2.

**Table 2.** Primers used in quantitative RT-PCR assay

Primer name	Primer sequences (5'-3')
H-FLI1-F	CCAACGAGAGGAGAGTCATCG
H-FLI1-R	TTCCGTGTTGTAGAGGGTGGT
H-Fibronectin-F	TTACCGTGGGCAACTCTGTC
H-Fibronectin-R	GTGTAGGGGTCAAAGCACGA
H-CollagenI-F	GCCAAGACGAAGACATCCCA
H-CollagenI-R	GGCAGTTCTTGGTCTCGTCA
hsa-miR-26a-RT	GTCGTATCCAGTGCAGGGTCCGAGGTATTCGCACTGGATACGACAGCCTA
hsa-miR-26a-F	GGGTTCAAGTAATCCAGGATA
mz-FLI1-F	ATGGACGGGACTATTAAGGAGG
mz-FLI1-R	GAAGCAGTCATATCTGCCTTGG
mz-Fibronectin-F	TTCAAGTGTGATCCCCATGAAG
mz-Fibronectin-R	CAGGTCTACGGCAGTTGTCA
mz-CollagenI-F	TTCTCCTGGCAAAGACGGAC
mz-CollagenI-R	CTCAAGGTCACGGTCACGAA
mmu-miR-26a-RT	GTCGTATCCAGTGCAGGGTCCGAGGTATTCGCACTGGATACGACAGCCTA
mmu-miR-26a-F	TTCAAGTAATCCAGGATA
U6-F	CTCGCTTCGGCAGCACA
U6-R	AACGCTTCACGAATTTGCGT
GAPDH-F	GAGTCAACGGATTTGGTCGT
GAPDH-R	GACAAGCTTCCCGTTCTCAG

### 2.4 Western blotting

Total proteins from cultured fibroblasts and mouse tissues were extracted using the ProteinExt® Mammalian Total Protein Extraction Kit (#DE101-01; TransGen Biotech; Beijing) following the manufacturer’s instructions. To extract protein from mouse tissues, the tissue samples were washed with cool PBS and homogenized in liquid nitrogen. To extract protein from cultured cells, cells were washed with PBS three times and then collected through centrifugation. After protein concentration was determined using the BCA method, approximately 30 μg protein of each sample was boiled at 100°C for 5 min in protein loading buffer. Proteins were then separated by 10% SDS-PAGE and transferred onto polyvinylidene difluoride (PVDF) membrane. After blocking with 5% skimmed milk solution for 2 h at room temperature, the membranes were then incubated with FLI1 primary antibody (#BA3239; Boster, 1:1000), collagen I primary antibody (#BA0325; Boster, 1:1000), Fibronectin primary antibody (#ab32419; Abcam, 1:1000), and GAPDH primary antibody (#10494-1-AP; Proteintech, 1:20,000) overnight at 4°C. Afterward, the membranes were washed with TBST three times for 10 min, followed by incubation with secondary antibodies (#EK1002, Boster, 1:1000). Finally, blots were developed using ECL western blotting detection reagents (#WB KLS0500; Millipore).

### 2.5 Target gene prediction and cell transfection

The target gene of the miRNA in this study was predicted using miRNA.org software package [25]. Mimics of miR-26a (5'-UUCAAGUAAUCCAGGAUAGGCU-3' and 5'-CCUAUCCUGGAUUACUUGAAUU-3'), miR-26a inhibitor (5'-AGCCUAUCCUGGAUUACUUGAA-3'), or respective negative controls, were all synthesized from the GenePharma Company (Suzhou, Jiangsu, China). SFBs were transfected with above mimics, inhibitor using the Lipofectamine™ 2000 (#11668019; Thermo Fisher Scientific) following the manufacturer’s protocol. The transfection efficiency was validated by determining miRNA expression using quantitative RT-PCR.

### 2.6 Drug-containing serum preparation

BSYJ was prepared as follows: 10 g *Asini Corii* Colla, 10 g *Cornus officinalis* Sieb. et Zucc., 20 g *Rehmannia glutinosa* (Gaertn.) DC., 30 g *Astragalus membranaceus* Moench, 30 g *Dioscorea japonica* Thunb., 15 g *Wolfiporia extensa* (Peck) Ginns, and 5 g *Carthamus tinctorius* Linn. were immersed together in 1.2 L distilled water for 20 min and boiled for 45 min. Then, the decoction was collected through filtration and was condensed into 134 mL through evaporation using water bath at 100°C. The dosage of obtained decoction was approximately 0.9 g/mL. Sprague–Dawley (SD) rats received the BSYJ (1.8 g/100 g) by intragastric administration twice daily for 3 days following with our previous study [10]. Blood was collected from the heart 3 h after the last drug administration under strict aseptic conditions, and serum was collected by centrifugation at 2500 rpm for 15 min and then stored at −80°C until further analysis.

### 2.7 Mouse *SSc* model and grouping

Twenty-five SPF C57BL/6 J mice aged 6–8 weeks were randomly divided into five groups (n = 5 in each group). After shaving hair from the area (1 × 1 cm) at the center of each mouse’s back, following treatments were given as indicated: normal control group (Control) was subcutaneously injected with 100 µL of normal saline daily combined with intragastric administration of normal saline (0.2 mL/10 g); model group (Model) was subcutaneously injected with 100 µL of 1 mg/mL bleomycin daily combined with intragastric administration of normal saline (0.2 mL/10 g); astragaloside group (AST) was subcutaneously injected with 100 µL of 1 mg/mL daily combined with intragastric administration of 4 mg/mL astragaloside; BSYJ group (BSYJ) was subcutaneously injected with 100 µL of 1 mg/mL bleomycin daily combined with intragastric administration of 0.2 mL/10 g BSYJ (the concentration of the BSYJ is 0.9 g/ml, which means the mice received 0.18 g/10 g BW BSYJ every day, which is equivalent to 15 mg/kg BW in human dose); miR-26a group (BSYJ + antagomir) was injected with 5 nM miR-26a antagomir every 3 days plus BSYJ treatment as above. Treatments were performed for 4 weeks, and mice were sacrificed for skin tissue collection near the injection points.

### 2.8 Masson’s trichrome and hematoxylin–eosin staining

Pathological alterations of mouse skin tissue were determined using Masson’s Trichrome Stain Kit (#G1340; Solarbio) according to the manufacturer’s instructions. Briefly, mouse skin tissue slides were subjected to conventional dewaxing to water, stained with Weigert’s iron hematoxylin for 5 to 10 min, incubated with acidic ethanol differentiation solution for 10 s, washed with water, incubated with Masson blue stain for 5 min, stained with Ponceau S-fuchsin for 5 min, rinsed with phosphomolybdic acid solution, stained with anilinum coeruleum for 1 min, dehydrated with 95% ethanol and 100% ethanal, cleared in xylene, and sealed with neutral balsam. Mouse skin tissues were also stained with hematoxylin–eosin. Tissue histological changes were finally observed under microscopy.

### 2.9 Statistics

Statistical significances between groups were analyzed using the SPSS 18.0 software. The differences between two groups were evaluated using Student’s *t*-test. The analysis of variance (ANOVA) was conducted to evaluate the significance among three groups, followed by post hoc Tukey, and *P* < 0.05 was considered to be statistically significant.

## 3. Results

The present study aims to investigate the underlying molecular mechanisms involved in the beneficial effects of BSYJ decoction on SSc. We hypothesized that FLI1 and miR-26a play important roles in BSYJ decoction’s protective functions against SSc. We validated our hypothesis using both SSc skin fibroblasts and a mouse model of SSc.

### 3.1 Fibroblast isolation and characterization

**Figure 1.**
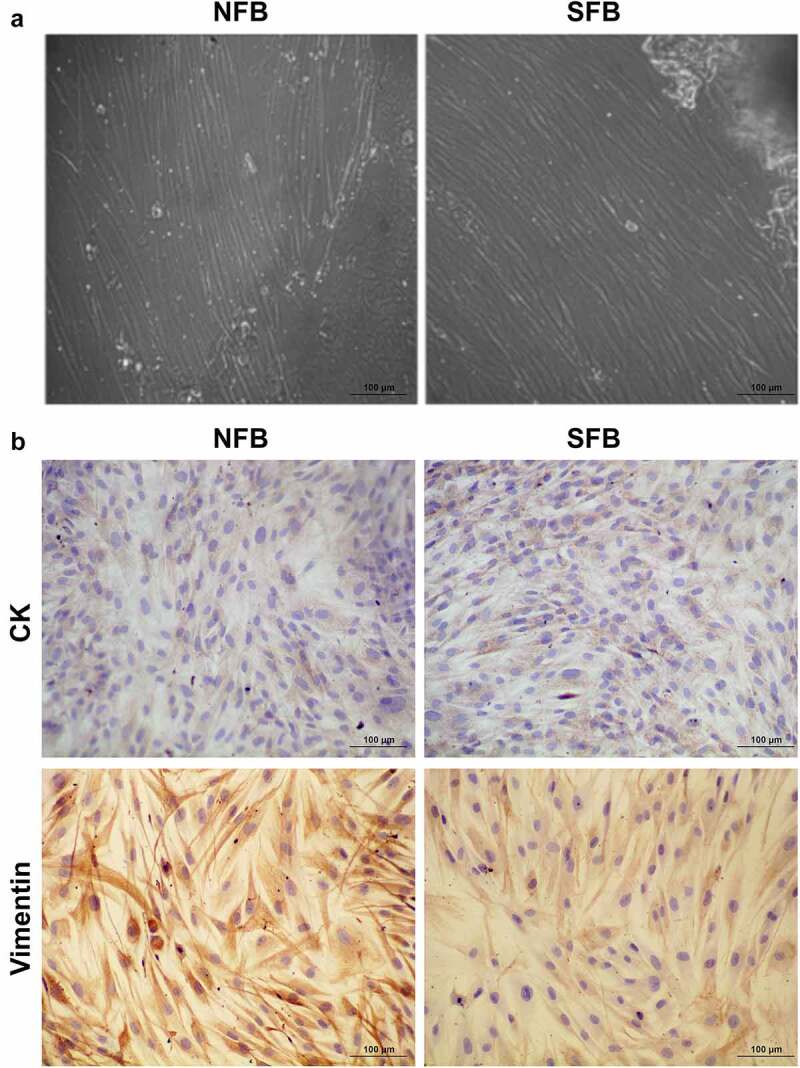
Primary human skin fibroblast culture and characterization

Primary cultures of normal skin fibroblasts (NFBs) and SFBs were established by a tissue-explant technique using skin tissues collected from healthy volunteers and SSc patients. The NFBs and SFBs under microscopy exhibited common morphological characteristics of human fibroblasts, as shown in Figure 1(a). We also detected the expression of two protein markers, cytokeratin (CK) and vimentin, in both primary NFBs and SFBs. We found that both primary NFBs and SFBs exhibited significant expression of vimentin. However, CK could not be detected (Figure 1(b)). The morphological characteristics and biomarker expression verified NFB and SFB as primary human fibroblasts which could be used for subsequent experiments.

(a) Morphological features of normal skin fibroblasts (NFBs) and SSc skin fibroblasts (SFBs) under conventional microscopy (magnification: 200×, scale bar: 100 μm). (b) Expression of cytokeratin (CK) and vimentin proteins in primary NFBs and SFBs. The protein expression in human skin fibroblasts was detected by immunocytochemistry using specific antibodies.

### 3.2 Decreased FLI1 expression in *SSc* skin fibroblasts

**Figure 2.**
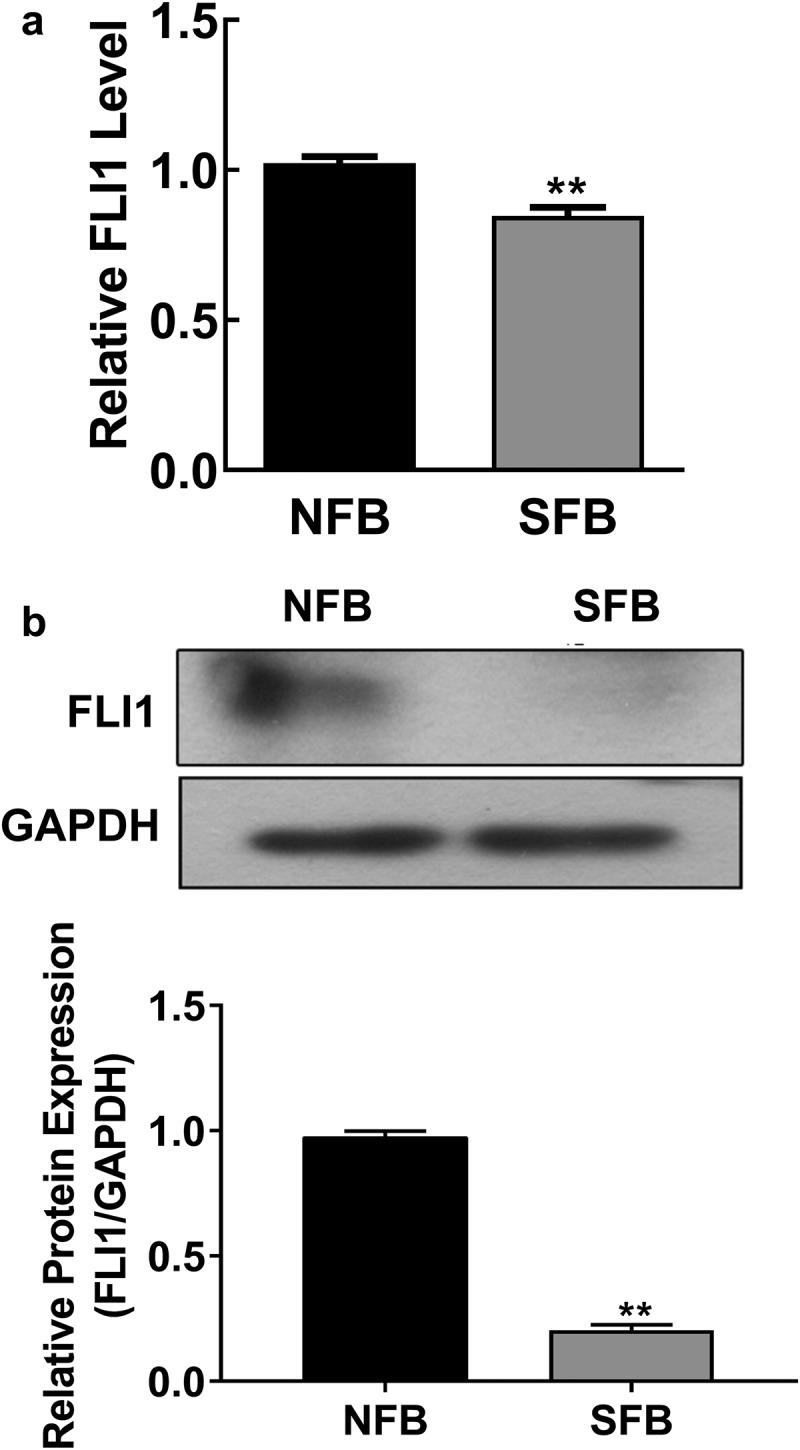
Downregulation of FLI1 expression in primary human skin fibroblast

Previous reports revealed that FLI1 gene expression decreased in skin fibroblasts during SSc pathogenesis, contributing to collagen expression and vascular homeostasis [4,5]. To further validate the role of FLI1 in SSc, we measured FLI1 gene expression in primary NFBs and SFBs. We found that the mRNA level of the FLI1 in primary SFBs was significantly lower than that in the primary NFBs (Figure 2(a)). Additionally, western blotting also showed a remarkable downregulation of FLI1 protein expression in primary SFBs compared with NFBs (Figure 2(b)). These results indicate the expressional change of the FLI1 is associated with SSc development.

(a) Relative FLI1 mRNA levels in NFBs and SFBs. The mRNA levels were measured by quantitative RT-PCR; glyceraldehyde-3-phosphate dehydrogenase (GAPDH) was used as an internal standard. (b) FLI1 protein abundance in NFBs and SFBs. FLI1 protein abundance was detected by western blotting; GAPDH was used as an internal standard. *N* = 3, ** *P* < 0.01.

### 3.3 Elevated miR-26a expression promotes *SSc* pathogenesis

**Figure 3.**
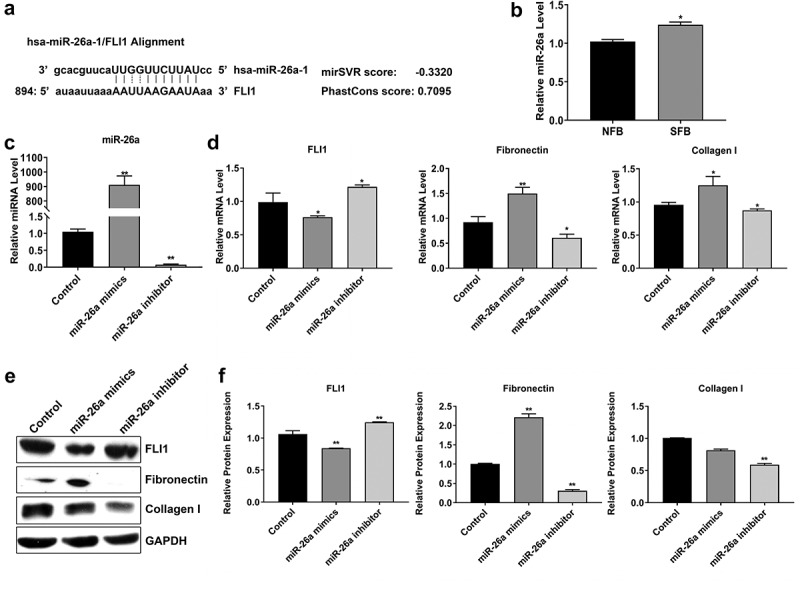
Suppression of FLI1 expression by miR-26a in SFBs

We predicted that miR-26a targets the 3' untranslated region (UTR) of FLI1 gene using the miRNA.org bioinformatics software (Figure 3(a)). To further explore the potential roles of miR-26a in SSc pathogenesis, miR-26a expression was measured in primary NFBs and SFBs. Using quantitative RT-PCR, we found that the miR-26a expression in SFBs was significantly higher than that in NFBs (Figure 3(b)). Furthermore, we manipulated miR-26a expression in SFBs by transfection with miR-26a mimics and inhibitor, respectively (Figure 3(c)). After elevating miR-26a expression in SFBs, FLI1mRNA expression was greatly repressed, whereas fibronectin collagen mRNA expression was significantly increased when compared with the control group (Figure 3(d)). On the contrary, when SFBs were transfected with miR-26a inhibitor, the mRNA expression of FLI1, fibronectin and collagen exhibited opposite modulation (Figure 3(d)). The changes of FLI1, fibronectin, and collagen expression in SFBs induced by miR-26a mimics and inhibitor were further validated by western blotting which showed consistent results as gene expression except collagen I protein expression was not significantly changed by miR-26a mimics (Figure 3(e,f)). These results demonstrate that miR-26a suppresses FLI1 and regulates fibrios-relevant modulator expression in SFBs.

(a) The association between miR-26a and 3'-UTR of the FLI1 gene was predicted by the miRNA.org software. (b) Relative miR-26a levels in NFBs and SFBs. MiRNA levels were analyzed by quantitative RT-PCR. (c) Relative miR-26a levels in SFBs transfected with miR-26a mimics and inhibitor. (d) Relative mRNA levels of FLI1, fibronectin, and collagen in SFBs transfected with miR-26a mimics and inhibitor. (e, f) Protein abundances of FLI1, fibronectin, and collagen in SFBs transfected with miR-26a mimics and inhibitor. Protein levels were determined by western blotting; GAPDH was used as the internal standard. *N* = 3, * *P* < 0.05; ** *P* < 0.01.

### 3.4 BSYJ suppresses SFB fibrosis by downregulation of miR-26a expression

**Figure 4.**
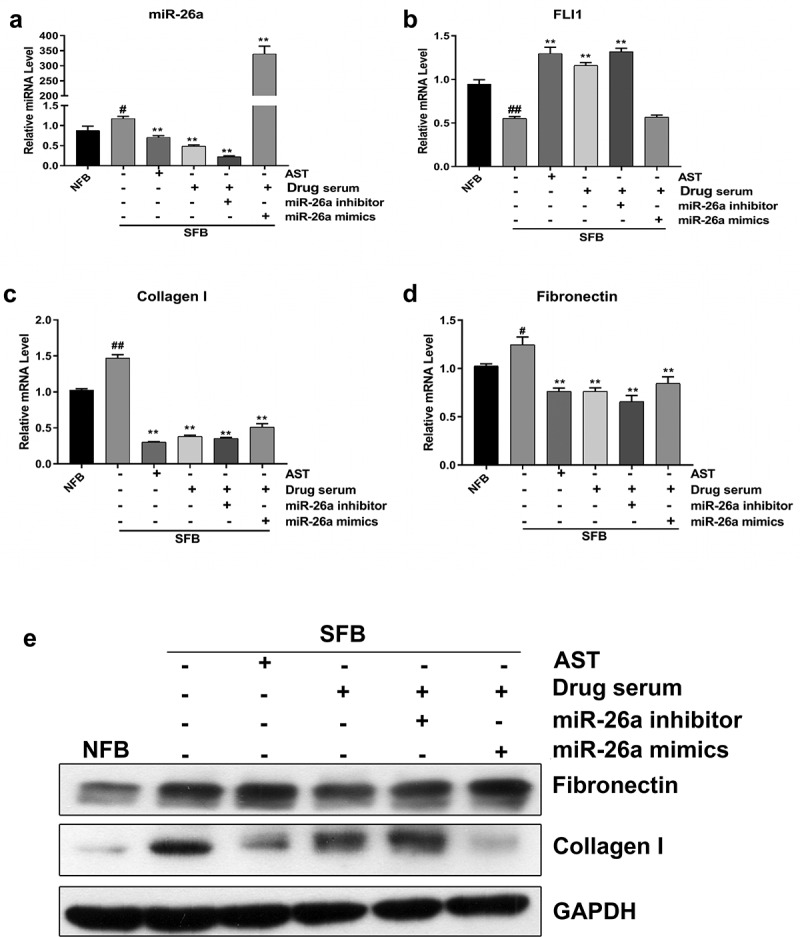
BSYJ suppresses SFB fibrosis *via* miR-26a-mediated FLI1 expression regulation

To study the therapeutic mechanism of BSYJ on SSc pathogenesis, the SFBs were treated with astragaloside (AST), BSYJ-containing rat serum, miR-26a mimics or inhibitor. We found that miR-26a expression in SFBs was significantly downregulated by either AST or BSYJ-containing rat serum treatment (Figure 4(a)). Consistently, FLI1 gene expression in SFBs was markedly elevated by treatment with either BSYJ-containing rat serum or AST (Figure 4(b)). Additionally, expression of collagen I and fibronectin in SFBs were greatly inhibited by treatment with either BSYJ-containing rat serum or AST (Figure 4(c,d)). The suppression of fibronectin and collagen I expression in SFBs by AST and BSYJ-containing rat serum was further verified by western blotting (Figure 4(e)). The remarkable alteration in FLI1 expression and expressional alterations of fibrosis marker genes (fibronectin and collagen I) in the BSYJ-containing serum-treated group indicated that BSYJ could suppress SSc pathogenesis by modulating the miR-26a-mediated FLI1 gene expression.

(a) Relative miR-26a levels in SFBs after treatment with astragaloside (AST), BSYJ-containing rat serum, miR-26a mimics or inhibitor. The miRNA levels were analyzed by quantitative RT-PCR. (b–d) Relative mRNA levels of FLI1 (b), collagen I (c), and fibronectin (d) in SFBs after treatment with AST, BSYJ-containing rat serum, miR-26a mimics and inhibitor by quantitative RT-PCR. (e) Abundance of fibronectin and collagen proteins in SFBs after treatment with AST, BSYJ-containing rat serum, miR-26a mimics, and inhibitor. Protein levels were analyzed by western blotting; GAPDH was used as the internal standard. *N* = 3, ^#^ and ^##^ indicate *P* < 0.05 and *P* < 0.01, respectively, compared with NFBs; * and ** indicate *P* < 0.05 and *P* < 0.01, respectively, compared with SFBs.

### 3.5 miR-26a silencing promotes BSYJ effects in mouse *SSc* model

**Figure 5.**
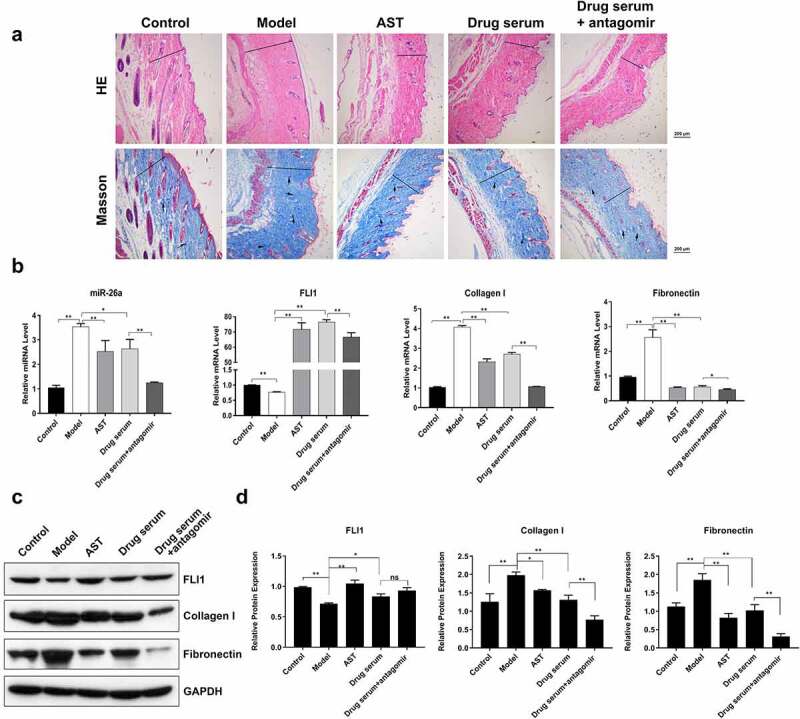
Enhanced anti-fibrosis effect of BSYJ by miR-26A suppression

To further validate the miR-26a-mediated anti-fibrosis effects of BSYJ, we established a mouse model of SSc which was induced by the injection of bleomycin. Masson’s trichrome and hematoxylin–eosin staining showed that collagen fiber deposition was greatly enhanced in the skin tissues of SSc mouse model when compared with the control group (Figure 5(a)). Treatment with AST and BSYJ significantly suppressed collagen fiber deposition in the skin of SSc mouse model (Figure 5(a)). Moreover, combination of BSYJ with antagomir which specifically inhibits miR-26a expression (Figure 5(b)) caused an even more significant inhibition of fibrosis in mouse skin tissue (Figure 5(a)). These results further indicated the important role of miR-26a in SSc pathogenesis and protective effects of BSYJ. Additionally, the expressions of fibronectin and collagen I in the skin tissue of SSc mouse model were also remarkably downregulated by combined application of BSYJ and antagomir when compared with BSYJ treatment alone (Figure 5(b)). The regulation of FLI1, fibronectin, and collagen I in SSc mouse model by combination of BSYJ with antagomir was further verified by western blotting results (Figure 5(c,d)). Together, our findings demonstrate that BSYJ suppresses fibrosis development during SSc pathogenesis through inhibiting miR-26a expression.

(a) Collagen fiber deposition in SSc mice after treatment with a combination of BSYJ and antagomir. Masson’s trichrome staining was performed to evaluate fiber accumulation and fibrosis in mice skin. The transverse line indicates the thickness of dermis and the arrow indicates collagen signal (magnification: 100×, scale bar: 200 μm). (b) Relative mRNA levels of FLI1, fibronectin, and collagen I and miR-26a in SSc mice skin treated with a combination of BSYJ and antagomir. Relative expression was analyzed by quantitative RT-PCR. (c, d) Protein abundance of FLI1, collagen I and fibronectin in SSc mice skin treated with a combination of BSYJ and antagomir, analyzed by western blotting. GAPDH was applied as the internal standard. *N* = 3, * and ** indicate *P* < 0.05 and *P* < 0.01, respectively.

## 4. Discussion

SSc is a complex pathogenic process associated with synergetic interaction of various signaling pathways and biological processes [3]. Excess accumulation of collagen fiber and resultant fibrosis are the characteristics of SSc pathogenesis and are attributed to expressional changes in a number of functional genes in fibroblasts [24,25]. The FLI1 gene functions as a key transcription factor which suppresses collagen I production in dermal fibroblasts, which was mediated by regulation of specificity protein 1 (Sp1)-dependent processes [26]. Previous investigation revealed that suppression of FLI1 expression or its function is a major mechanism contributing to collagen deposition and dermal fibrosis during SSc pathogenesis [4,5,29]. Additionally, epigenetic regulations such as DNA methylation and histone deacetylation at the FLI1 gene modulate its expression in skin tissues of SSc [5]. In the present study, we isolated primary fibroblasts from both healthy volunteers and SSc patients and found that FLI1 gene expression was significantly suppressed in SFBs. These results further support the relationship between FLI gene expression and fibrosis during SSc pathogenesis. However, molecular mechanisms of FLI1 suppression during SSc pathogenesis remain poorly understood.

FLI1 gene expression was reported to be modulated by miRNAs in certain contexts. For example, microvascular-selective miRNAs, such as miR-145 expressed in pericytes inhibits FLI1 expression to regulate microvascular cell migration in response to growth factors [28]. In this study, we used bioinformatics to predict FLI1 is a target gene of miR-26a. MiR-26a participates in various biological and pathogenic processes, such as autoimmune diseases, including multiple sclerosis (MS), influenza virus infection, and cancer progression [30–33]. A previous study revealed that miR-26a expression was also downregulated in hepatocellular carcinoma cells, and increased expression of miR-26a in liver cancer cells led to inhibition of cancer cell proliferation, tumor-specific apoptosis, and cell cycle arrest by directly targeting cyclin proteins [34]. MiR-26a was found to suppress cell growth and tumor development by inhibiting the expression of the enhancer of zeste homolog 2 (EZH2) accounting for the tumorigenesis of nasopharyngeal carcinoma [35]. Other genes targeted by miR-26a include SMAD1 which is related to osteogenic differentiation of human adipose tissue stem cells [36], and PTEN which is related to metastases in lung cancer cells [37]. miR-26a targets various genes in different pathogenic processes clearly demonstrates its great variability and significance of miR-26a in the development of human diseases.

Despite the close association of miR-26a with fibrosis-related disorders, such as idiopathic pulmonary fibrosis and cardiac fibrosis, little is known about the role of miR-26a in the pathogenic processes of SSc [21,22]. In this study, we demonstrated that miR-26a expression was greatly elevated in both primary SFBs and a mouse model of SSc. Alteration of miR-26a expression in SFB cells by transfection with miR-26a mimics or inhibitor resulted in a negative regulation of FLI1 gene expression in SFBs. This further supports that the FLI1 gene is a target of miR-26a in SFBs. Furthermore, the suppression of FLI1 expression by miR-26a was also associated with fibrosis progression during the development of SSc, which is supported by the findings that fibronectin gene and protein expressions were upregulated or downregulated by miR-26a mimics and inhibitor, respectively. MiR-26a inhibitor suppressed both collagen I gene and protein expression, while the expression change of collagen I is different between the gene levels and protein levels in miR-26a mimics group. The discrepancy between collagen I gene and protein expression might be associated with complicated and varied post-transcriptional mechanisms involved in turning mRNA into protein, which is frequently observed in research [38]. Collagen I has been established as a main component of overexpressed extracellular matrix proteins during dermal fibrosis and SSc pathogenesis [27]. Fibronectin is another member of extracellular matrix proteins, which is produced by fibroblasts and over-accumulated during fibrosis progression in SSc patients [39]. The significant regulation of both collagen I and fibronectin gene expression in SFBs by miR-26a convincingly supports the hypothesis that miR-26a functions as a key player during fibrosis progression which is associated with SSc pathogenesis.

We subsequently revealed that serum from rats treated with BSYJ could repress miR-26a expression, resulting in recovery of FLI1 gene expression and decreased accumulation of collagen I and fibronectin in SFBs. These results suggest that miR-26a-suppressed FLI1 gene expression is involved in the beneficial effects of BSYJ treatment on inhibition of fibrosis progression. We further confirm our finding *in vivo* using a mouse model of SSc. We found that BSYJ significantly repressed fibrosis symptoms in mice by modulation of miR-26a, FLI1, collagen I, and fibronectin gene expression in the skin tissue. Astragaloside is a bioactive component derived from *Astragalus membranaceus* and a constituent of BSYJ. It has been reported to suppress progression of various fibrosis processes, such as renal fibrosis and bleomycin-induced pulmonary fibrosis [40,41]. In this study, we also revealed that AST could induce remarkable alteration of miR-26a, FLI1, collagen I, and fibronectin expression in SFBs and skin tissues of the mouse models. It also suppresses fiber deposition and fibrosis progression in mice with SSc. These results provide a preliminary explanation of the chemical basis of the anti-SSc effects of BSYJ. Other bioactive chemicals in BSYJ, such as hydroxysafflor yellow A from *Carthamus tinctorius*, also inhibited fibrosis under certain conditions, suggesting that they have concordant effects on SSc progression [40]. The functions of other bioactive components in the BSYJ decoction on SSc progression are worth further investigation.

## 5. Conclusion

To conclude, our study demonstrates that miR-26a is upregulated during SSc progression, which promotes dermal fibrosis by targeting the FLI gene and extracellular matrix protein deposition in primary human SFBs and in the mouse model of SSc. More importantly, the miR-26a-mediated FLI1 suppression was associated with the anti-SSc effects of the BSYJ decoction. Our findings provide insight into the potential use of traditional medicine and miRNA-mediated epigenetic regulation as a treatment for SSc.

## References

[1] Fett N. Scleroderma: nomenclature, etiology, pathogenesis, prognosis, and treatments: facts and controversies. Clin Dermatol. 2013;31(4):432–437. 23806160 10.1016/j.clindermatol.2013.01.010

[2] Gilbane AJ, Denton CP, Holmes AM. Scleroderma pathogenesis: a pivotal role for fibroblasts as effector cells. Arthritis Res Ther. 2013;15(3):215. 23796020 10.1186/ar4230 PMC4060542

[3] Denton CP, Black CM, Abraham DJ. Mechanisms and consequences of fibrosis in systemic sclerosis. Nat Clin Pract Rheumatol. 2006;2(3):134. 16932673 10.1038/ncprheum0115

[4] Asano Y, Stawski L, Hant F, et al. Endothelial Fli1 deficiency impairs vascular homeostasis: a role in scleroderma vasculopathy. Am J Pathol. 2010;176(4):1983–1998. 20228226 10.2353/ajpath.2010.090593 PMC2843486

[5] Wang Y, Fan PS, Kahaleh B. Association between enhanced type I collagen expression and epigenetic repression of the FLI1 gene in scleroderma fibroblasts. Arthritis Rheum. 2006;54(7):2271–2279. 16802366 10.1002/art.21948

[6] Qi Q, Mao Y, Yi J. Effect of Bushen Yijing Formula on dermal fibrosis in scleroderma model mice. J New Chin Med. 2012;6:149–151.

[7] Qi Q, Mao Y, Yi J. Research on Bushen Yijing Therapy to the balance of SMAD3/FLI1 in fibroblast of scleroderma. J Sichuan Trad Chin Med. 2012;30(6):43–44.

[8] Qiu LP, Tian YZ, Zhang JL, et al. Effects of Bushen Yijing therapy on endothelial to mesenchymal transition in systemic scleroderma. J Shanghai Univ Trad Chin Med. 2017;31(1):63–68.

[9] Han J, Denf W, Xie S. Effect of tonifying kidney and strengthening essence methods on endothelial-mesenchymal transition scleroderma mice based on regulation of Fli1. Global Trad Chin Med. 2018;11(10):19–24.

[10] Tong PD, Wang ML, Hu X, et al. Effect of retarding aging Chinese drug Bushen Yijing Decoction on rat left ventricular myosin heavy chain(MHC) mRNA Change with aging. Chin J Basic Med Tradit Chin Med. 2001;7(4):34–36.

[11] Zhang G, Ma J, Zhang Q. [Experimental study on effect of Bushen Yijing recipe in delaying senility of bone and brain of aged male rats]. Zhongguo Zhong Xi Yi Jie He Za Zhi. 2000;20(1):43–45. 11783337

[12] Zhu TY, Shi YY, Zhang G. Experimental study of reinforcing effect of Bushen Yijing capsule on bone quality of cancellous bone in ovariectomy caused rats osteoporosis model. Zhongguo Zhong Xi Yi Jie He Za Zhi. 2001;21(9):688–691. 12575559

[13] Kim DH, Saetrom P, Snove SO, et al. MicroRNA-directed transcriptional gene silencing in mammalian cells. Proc Natl Acad Sci U S A. 2008;105(42):16230–16235. 18852463 10.1073/pnas.0808830105 PMC2571020

[14] Saj A, Lai EC. Control of microRNA biogenesis and transcription by cell signaling pathways. Curr Opin Genet Dev. 2011;21(4):504–510. 21592778 10.1016/j.gde.2011.04.010 PMC3149747

[15] O’Reilly S. MicroRNAs in fibrosis: opportunities and challenges. Arthritis Res Ther. 2016;18(1):11. 26762516 10.1186/s13075-016-0929-x PMC4718015

[16] Henderson J, Distler J, O’Reilly S. The role of epigenetic modifications in systemic sclerosis: a druggable target. Trends Mol Med. 2019;25(5):395–411. 30858032 10.1016/j.molmed.2019.02.001

[17] Zhu H, Li Y, Qu S, et al. MicroRNA expression abnormalities in limited cutaneous scleroderma and diffuse cutaneous scleroderma. J Clin Immunol. 2012;32(3):514–522. 22307526 10.1007/s10875-011-9647-y

[18] Etoh M, Jinnin M, Makino K, et al. MicroRNA-7 down-regulation mediates excessive collagen expression in localized scleroderma. Arch Dermatol Res. 2013;305(1):9–15. 22965811 10.1007/s00403-012-1287-4

[19] Zhu H, Luo H, Li Y, et al. MicroRNA-21 in scleroderma fibrosis and its function in TGF-β-regulated fibrosis-related genes expression. J Clin Immunol. 2013;33(6):1143–1144. 10.1007/s10875-013-9896-z 23657402

[20] Makino T, Jinnin M, Etoh M, et al. Down-regulation of microRNA-196a in the sera and involved skin of localized scleroderma patients. Eur J Dermatol. 2014;24(4):470–476. 25152444 10.1684/ejd.2014.2384

[21] Liang H, Xu C, Pan Z, et al. The antifibrotic effects and mechanisms of microRNA-26a action in idiopathic pulmonary fibrosis. Mol Ther. 2014;22(6):1122–1133. 24594795 10.1038/mt.2014.42 PMC4048895

[22] Wei C, Kim IK, Kumar S, et al. NF-κB mediated miR-26a regulation in cardiac fibrosis. J Cell Physiol. 2013;228(7):1433–1442. 23254997 10.1002/jcp.24296

[23] Su N, Qian M, Deng M. Integrative approaches for microRNA target prediction: combining sequence information and the paired mRNA and miRNA expression profiles. Curr Bioinform. 2013;8(1):37–45. 23467572 10.2174/1574893611308010008 PMC3583062

[24] Balbir-Gurman A, Braun-Moscovici Y. Scleroderma - new aspects in pathogenesis and treatment. Best Pract Res Clin Rheumatol. 2012;26(1):13–24. 22424190 10.1016/j.berh.2012.01.011

[25] Ponticos M, Papaioannou I, Xu S, et al. The failure to degrade JunB contributes to Collagen type I over-production and dermal fibrosis in Scleroderma. Arthritis Rheumatol. 2014;67(1):243–253. 10.1002/art.38897 PMC4312903 25303440

[26] Czuwaraladykowska J, Shirasaki F, Jackers P, et al. Fli-1 inhibits collagen type I production in dermal fibroblasts via an Sp1-dependent pathway. J Biol Chem. 2001;276(24):20839–20848. 11278621 10.1074/jbc.M010133200

[27] Noda S, Asano Y, Nishimura S, et al. Simultaneous downregulation of KLF5 and Fli1 is a key feature underlying systemic sclerosis. Nat Commun. 2014;5(6):5797. 25504335 10.1038/ncomms6797 PMC4268882

[28] Larsson E, Fuchs PF, Heldin J, et al. Discovery of microvascular miRNAs using public gene expression data: miR-145 is expressed in pericytes and is a regulator of Fli1. Genome Med. 2009;1(11):108. 19917099 10.1186/gm108 PMC2808743

[29] Liu H, Song L, Huang W. MiR26a and miR939 regulate the replication of H1N1 influenza virus in MDCK cell. Wei Sheng Wu Xue Bao. 2010;50(10):1399–1405. 21141477

[30] Sekimoto N, Suzuki A, Suzuki Y, et al. Expression of miR‑26a exhibits a negative correlation with HMGA1 and regulates cancer progression by targeting HMGA1 in lung adenocarcinoma cells. Mol Med Rep. 2017;15(2):534–542. 28000891 10.3892/mmr.2016.6053 PMC5364888

[31] Zhang R, Tian A, Wang J, et al. MiR26a modulates Th17/T reg balance in the EAE model of multiple sclerosis by targeting IL6. Neuromol Med. 2015;17(1):24–34. 10.1007/s12017-014-8335-5 25362566

[32] Kota J, Chivukula RR, O’Donnell KA, et al. Therapeutic delivery of miR-26a inhibits cancer cell proliferation and induces tumor-specific apoptosis. Cell. 2009;137(6):1005.19524505

[33] Lu J, He M, Wang L, et al. MiR-26a inhibits cell growth and tumorigenesis of nasopharyngeal carcinoma through repression of EZH2. Cancer Res. 2011;71(1):225–233. 21199804 10.1158/0008-5472.CAN-10-1850

[34] Luzi E, Marini F, Sala SC, et al. Osteogenic differentiation of human adipose tissue-derived stem cells is modulated by the miR-26a targeting of the SMAD1 transcription factor. J Bone Miner Res. 2010;23(2):287–295. 10.1359/jbmr.071011 18197755

[35] Liu B, Wu X, Liu B, et al. MiR-26a enhances metastasis potential of lung cancer cells via AKT pathway by targeting PTEN. Biochim Biophys Acta. 2012;1822(11):1692–1704. 22885155 10.1016/j.bbadis.2012.07.019

[36] Wang D. Discrepancy between mRNA and protein abundance: insight from information retrieval process in computers. Comput Biol Chem. 2008;32(6):462–468. 18757239 10.1016/j.compbiolchem.2008.07.014 PMC2637108

[37] Kinsella MB, Smith EA, Miller KS, et al. Spontaneous production of fibronectin by alveolar macrophages in patients with scleroderma. Arthritis Rheum. 2014;32(5):577–583. 10.1002/anr.1780320511 2719731

[38] Che X, Wang Q, Xie Y, et al. Astragaloside IV suppresses transforming growth factor-β1 induced fibrosis of cultured mouse renal fibroblasts via inhibition of the MAPK and NF-κB signaling pathways. Biochem Biophys Res Commun. 2015;464(4):1260–1266. 26220342 10.1016/j.bbrc.2015.07.116

[39] Qian W, Cai X, Qian Q, et al. Astragaloside IV modulates TGF‐β1‐dependent epithelial‐mesenchymal transition in bleomycin‐induced pulmonary fibrosis. J Cell Molr Med. 2018;22(9):4354–4365. 10.1111/jcmm.13725 PMC6111865 29971947

[40] Wang CY, Liu Q, Huang QX, et al. Activation of PPARγ is required for hydroxysafflor yellow A of Carthamus tinctorius to attenuate hepatic fibrosis induced by oxidative stress. Phytomedicine. 2013;20(7):592–599. 23523101 10.1016/j.phymed.2013.02.001

